# Dietary phytate induces subclinical mechanical allodynia in mice

**DOI:** 10.1590/1414-431X2023e12955

**Published:** 2023-11-03

**Authors:** D.O. Matias, T. Sisnande, A.F. Martins, M.J. do Amaral, B.L.R. Santos, A.L.P. Miranda, L.M.T.R. Lima

**Affiliations:** 1 Laboratório de Biotecnologia Farmacêutica, Faculdade de Farmácia, Universidade Federal do Rio de Janeiro, Rio de Janeiro, RJ, Brasil; 2 Laboratório de Estudos em Farmacologia Experimental, Faculdade de Farmácia, Universidade Federal do Rio de Janeiro, Rio de Janeiro, RJ, Brasil; 3 Programa de Pós-Graduação em Química Biológica, Universidade Federal do Rio de Janeiro, Rio de Janeiro, RJ, Brasil; 4 Programa de Pós-Graduação em Ciências Farmacêuticas, Universidade Federal do Rio de Janeiro, Rio de Janeiro, RJ, Brasil; 5 Laboratório de Macromoléculas, Instituto Nacional de Metrologia, Qualidade e Tecnologia, Duque de Caxias, RJ, Brasil

**Keywords:** Phytate, Allodynia, Pain, Micronutrient, Inflammation

## Abstract

Neuropathic pain is a condition with varying origins, including reduced dietary micronutrient intake. Phytate is a polyphosphate found in seeds and grains that can act as an antinutrient due to the ability of sequester essential divalent metals. Here we tested whether moderate dietary phytate intake could alter nociceptive pain. We subjected weaning mice to a chow supplemented with 1% phytate for eight weeks. Body weight gain, glycemic responses, food ingestion, water ingestion, and liver and adipose tissue weights were not altered compared to controls. We observed a decreased mechanical allodynia threshold in the intervention group, although there were no changes in heat- or cold-induced pain. Animals consuming phytate showed reduced spinal cord tumor necrosis factor (TNF), indicating altered inflammatory process. These data provide evidence for a subclinical induction of mechanical allodynia that is independent of phytate consumption in animals with otherwise normal phenotypic pattern.

## Introduction

Pain is an unpleasant experience that indicates maladies in the organism due to various causes including injuries (1), trauma, metabolic dysfunction, pathogenic infections, cancer, and other idiopathic causes (2).

Three classes of pain are known. Nociceptive pain is related to stimuli with some protective function. Inflammatory pain is associated to tissue damage that requires cell repair with immune cell infiltration and may persist until the damage is repaired. Pathologic pain is related to damage to the nervous system (neuropathic pain) or dysfunction (dysfunctional pain) (3).

It has been argued that micronutrient deficiency (such as Zn, Mg, Ca, Cu) may result in the development and worsening of chronic pain (4). We have recently shown that dietary restriction of zinc results in endocrine degeneration of the pancreas resembling non-autoimmune type 1 diabetes (5). This moderate dietary restriction of zinc ions resulted in the development of mechanical allodynia and the expression of inflammatory biomarkers, including tumor necrosis factor (TNF) and superoxide dismutase (SOD), (6), indicating that zinc deficiency alone may result in the deflagration of nociceptive pain.

Mineral deficiencies may occur from the consumption of foods low in micronutrients (7,8) and from the consumption of anti-nutrients such as phytate. Phytate (myo-inositol (1-6) hexaphosphate) is a major compound found in seeds and grains. While phytate is an important natural source for phosphorous (P), it can decrease the bioavailability of micronutrients, particularly divalent ions (such as Fe^2+^, Zn^2+^, Cu^2+^, Mg^2+^, and Ca^2+^), by sequestering these essential metals hampering their gastro-intestinal absorption (9).

In this context, we designed a study aiming to evaluate the effect of dietary phytate on the development of pain in mice.

## Material and Methods

### Material

Phytic acid was obtained from Galena (Galena Química e Farmacêutica Ltda., Brazil; CAS 83-86-3; Cat #02037630102-C_6_H_18_O_24_P_6_, MW 660.04). Sodium phytate was obtained by neutralizing 10 g of phytic acid solution (equivalent to 7.37 g phytic acid and 2.01 g phosphorous) with 6 M NaOH until pH 7.0 (each 10 mL phytic acid solution required consuming about 19 mL 6 M NaOH, equivalent to about 2.62 g Na^+^), followed by lyophilization.

### Animals

This study used 21-day-old male Swiss mice, housed at 22±2°C, 60-80% humidity, and a 12-h light/dark cycle in the vivarium of the Faculty of Pharmacy (UFRJ). Animals had free access to water and food. After weaning, animals were randomized into the two study arms, and received the control diet or the intervention diet (phytate diet), with 5 to 6 animals per group. The present study was conducted with approval of the Animal Care Committee of the Federal University of Rio de Janeiro (CEUA-UFRJ) (protocol CCS-UFRJ-086/2021).

### Rodent diet

We used standard rodent chow (Labgold RC20 mm - 6554, Socil^®^, Brazil). Pellets were pulverized with an industrial blender, and in the intervention diet, the chow was mixed with powdered sodium phytate at 10 g phytate/kg chow (1% w/w phytate, excluding sodium). The amount of phytate was confirmed by mineral analysis (Table 1). The control group received the chow without further modification, since compensation for the added sodium alone was not possible without the introduction of other anions as variables.

**Table 1:** Mineral content analysis of diets.

Mineral	Diet		Change (%)
	Control (mg/kg)	Phytate (mg/kg)	
Sodium	1,606.4	4,684.1	291
Phosphorous	8,404.6	10,678.9	127

### Experimental design

**Figure 1:**
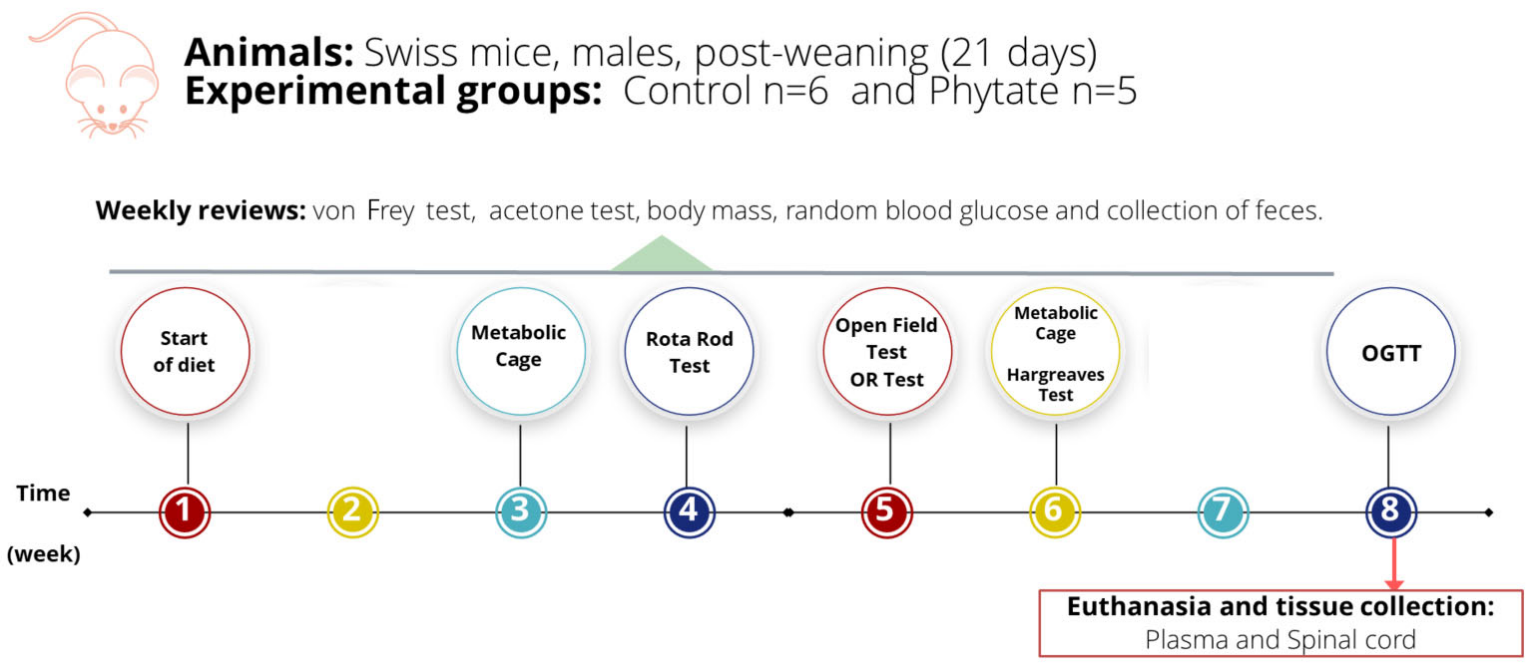
Experimental design. Animals were weaned onto their respective diet (control, n=6; supplemented with phytate, n=5) until euthanasia after 8 weeks. Animals were tested weekly for body mass, capillary glycemia, and mechanical and cold allodynia. The rota-rod test was conducted in the 4th week of intervention, and the metabolic cage assessment was conducted in the 3rd and 6th weeks of intervention. In the 5th week, animals were tested for object recognition (OR), open field, Hargreaves test, and oral glucose tolerance test (OGTT).

The summary of the study design is presented in Figure 1. Animals were weaned onto their corresponding diet (control/phytate) according to the randomization for 8 weeks before euthanasia.

Animals were evaluated weekly for their body mass, capillary glycemia, and mechanical (von Frey test) and cold (acetone) allodynia. Feces were collected throughout the intervention, combined according to the group, and stored at -20°C for further mineral content analysis.

Food and water consumption and feces and urine production were accessed by the metabolic cage at weeks 3 and 6 of the study, as described below.

Hargreaves's thermal sensitivity test was performed on week 6 after weaning.

Motor capacity (rota-rod test) and anxiety behavior (open field test) were evaluated at weeks 4 and 5, respectively. Exploratory and memory capacities were evaluated at week 8.

Oral glucose tolerance test was evaluated at week 8 of the intervention.

At termination, biological specimens were collected, prepared for histological analysis, and/or stored at -80°C for further analysis.

### Metabolic cage

The evaluation of food and water consumption and the production of feces and urine were performed using the single-mouse metabolic cage (Cat #3600M021; Tecniplast, Italy) for 12 h during dark cycle, at 22±2°C and 60-80% relative humidity. Food and water were measured before and after the intervention, and the difference represented their consumption. Feces and urine were collected and quantified, and the feces were stored at -20°C for further mineral analysis, as previously mentioned.

### Mineral analysis

The feces were collected and combined according to the randomization group, stored at -20°C, and further lyophilized (Liotop 101; Liobras^®^, Brazil) for 72 h.

The humidity of the feces was 63.1% in the control group and 59.6% in the intervention group. No error could be estimated for this measure since the feces were combined throughout the 8 weeks intervention into a single batch according to the group due to the high amount of material required for mineral analysis.

The dry feces as well as the diets (control and intervention) used in the study were subjected to total mineral content analysis (Table 2) by the Brazilian Agricultural Research Corporation (EMBRAPA Food Technology, Brazil) by inductively coupled plasma - optical emission spectrometry (ICP-OES) according to the internal Standard Operational Protocol (Min-026-rev0). According to the analysis report, the lower limit of detection (LLOD) was 0.01 mg/kg and the lower limit of quantification (LLQ) was <0.10 mg/kg.

**Table 2:** Mineral fecal content of control and phytate-supplemented diets.

Mineral	Diet		Change (%)
	Control (g/kg)	Phytate (g/kg)	
Sodium	4.22	5.09	+20.6
Potassium	6.75	6.12	-9.3
Magnesium	7.94	7.97	+0.34
Calcium	29.32	30.43	+3.8
Manganese	0.427	0.415	-2.8
Iron	1.74	1.22	-29.8
Zinc	0.313	0.306	-2.2
Copper	0.0669	0.0637	-4.78
Phosphorous	17.62	19.11	+8.4
Aluminum	0.251	0.238	-5.2

### Motor capacity

The motor capacity (balance/coordination) was accessed using the Rota Rod apparatus (EF412, Insight^®^, Brazil) (10,11). The test was performed using progressive acceleration from 4 to 40 rpm in 300 s. The number of falls and latency were electronically detected and reported. Previous adaptation to the apparatus was performed once a day for the three days before testing, where animals had three attempts to stay in the equipment. In the evaluation day, the mice were maintained in their respective cages in the test room for 15 min before the test.

### Animal behavior - open field test (OFT)

The open field test (OFT) was conducted before the object recognition test (ORT) (12,13). In the OFT, the mouse was placed in a wood apparatus (60×40×50 cm^3^) with 12 quadrant divisions (15.0×13.3 cm^2^ each) in the base of the apparatus. The animal was evaluated for 5 min concerning the following parameters: number of crossings between divisions, total travelled distance, time in the center and in the periphery of the box, latency, and exploration time. A camera in the upper part of the apparatus recorded the experiment, and the parameters were evaluated and quantified using an automatic tracking system (ANY-Maze, https://www.any-maze.com/) (14).

### Object recognition test

The declarative memory was accessed by the ORT, based on the natural tendency of the mouse to spend more time exploring a new object than an already known one (15,16). The test was performed in two steps, named training and evaluation. In the first step, the animal was placed in the same apparatus used in the OFT described above, containing two objects of similar dimensions and color, and allowed 5 min to explore the objects. After 60 min, a new test was performed in which one of the two objects was replaced with a new one with different dimensions and color, and the animal was allowed to explore for five minutes. The results are reported as the time exploring each object in the training and the evaluation steps.

### Mechanical allodynia - von Frey test

The evaluation of mechanical allodynia was conducted using the von Frey test with the “up-down” method (17). The animals were individually placed in acrylic boxes (9×7×11 cm^3^) with a metallic mesh as the floor, and animal's paws were stimulated with calibrated filaments (0.008 to 2.0 g; Bioseb Lab Instr, Cat #BIO-VF-M). After adaptation to the cage, the animals were tested with varying filaments starting at 0.6 g for 5 times with 60 s intervals between stimuli, and higher or lower strengths were applied according to the response until convergence of response (three withdrawals out of five stimuli).

### Heat allodynia - Hargreaves test

Thermal sensitivity was evaluated using the Hargreaves test (18). Animals were placed on a glass surface for 20 min for adaptation, followed by an increase in temperature from 30 to 55°C (with a rate of 1 to 2°C/s) using a tungsten lamp. Stimulation was interrupted upon paw withdrawal and the time between turning the lamp on and off was reported as the time for response to nociceptive stimulation.

### Cold allodynia - acetone test

Cold sensitivity was accessed by the acetone test (19,20). The animals were individually placed in acrylic boxes (9×7×11 cm^3^) over a metallic mesh allowing access to animal's paws. After adaptation for 30 min, 100 μL acetone was dispensed on the plantar surface of the mouse with the aid of a syringe. The animals were observed for up to 2 min, and the licking and/or flapping time in response to acetone was registered and used as thermal sensitivity to cold.

### Capillary glycemia

The mice were screened for their random (no fasting) capillary glycemia (21) using a digital glucometer (AccuChek Active^®^, Roche^®^, Brazil) from the tail tip after a small incision (about 1 mm), and the measured glycemia (in mg/dL) was recorded.

### Oral glucose tolerance test (OGTT)

The resistance to glucose was assessed by monitoring the glycemic response upon an oral glucose load of 2 g/kg body weight (21,22). The baseline glycemia was measured after 6 h of fasting, followed by gavage with a freshly prepared of 30% (w/v) glucose solution. Further measurements were performed at 15, 30, 60, 90, and 120 min after oral glucose load. Glucose (mg/dL) was measured in caudal blood collected directly on the ribbon of the Roche^®^ AccuChek active blood glucose meter.

### Immunoassay (ELISA) for TNF

The tissue levels of TNF in the lumbar spinal cord were determined by TNF ELISA from BD Pharmingen (Purified Hamster Anti-Mouse/Rat TNF, Cat #557516; detection antibody Biotin Human anti-Mouse/Rat TNF, Cat #558415; Standard TNF was recombinant mouse TNF, Cat #554589; BD Pharmingen; MaxSorb 96 multiwell plate, Nunc^®^, USA). The tissue was homogenized with a Bullet Blender^®^ (Next Advance, USA) in polypropylene tubes (Axygen^®^, nominal capacity 1.5 mL, USA) with extraction buffer (100 mM sodium phosphate, 1 mM de EDTA, 10 mM indomethacin, pH 7.4) in the proportion of 100 µL buffer/10 mg tissue and using 1.0 mm zirconium oxide beads (ZROB10). Trituration was performed for 3 cycles of 5 min with 1 min intervals in ice. The samples were further centrifuged for 10 min at 14,000 *g* and 4°C, and the total protein was quantified by the Bradford method using albumin as standard. The results are reported as pg TNF/mg total tissue protein.

### Plasma biochemical analysis

After euthanasia, the blood was collected by cardiac puncture in EDTA, and the plasma was obtained by centrifugation (10,000 *g*; 10 min; 4°C). Plasma aliquots were subjected to biochemical analysis for lipid fractions (total cholesterol, high-density lipoprotein cholesterol (HDLc), low-density lipoprotein cholesterol (LDLc), very-low-density lipoprotein cholesterol (VLDL), and triglycerides), enzymes lactate dehydrogenase (LDH), aspartate aminotransferase (AST), alanine aminotransferase (ALT), and total plasma protein, serum albumin, and globulin. All analyses were automated in a Beckman AU5800 (Beckman Coulter Diagnostics, USA; from the Hospital Universitario Clementino Fraga Filho (HUCFF), UFRJ according to internal standardization.

### Statistical analysis

All results are reported as mean and standard deviation. The normality of the data was tested using Kolmogorov Smirnov, D'Agostino, and Shapiro-Wilk tests. The distribution normality was evaluated using one-way or two-way analysis of variance (ANOVA) with repeated measurements, followed by Sidak's and Bonferroni post-tests. Data without normal distribution were subjected to Student's *t*-test or Wilcoxon analysis. P-values lower than 0.05 were considered significant. The statistical analysis was conducted using GraphPad Prism^®^ 7.00 (USA).

## Results

### Nutrient consumption was not affected by the presence of phytate

The control and the intervention diets were analyzed for nutrient content. A higher content of sodium (291%) and phosphorous (127%) was found in the intervention diet compared to control diet, consistent with the presence of phytate (Table 1).

The results of the micronutrient content of feces are shown in Table 2.

**Figure 2:**
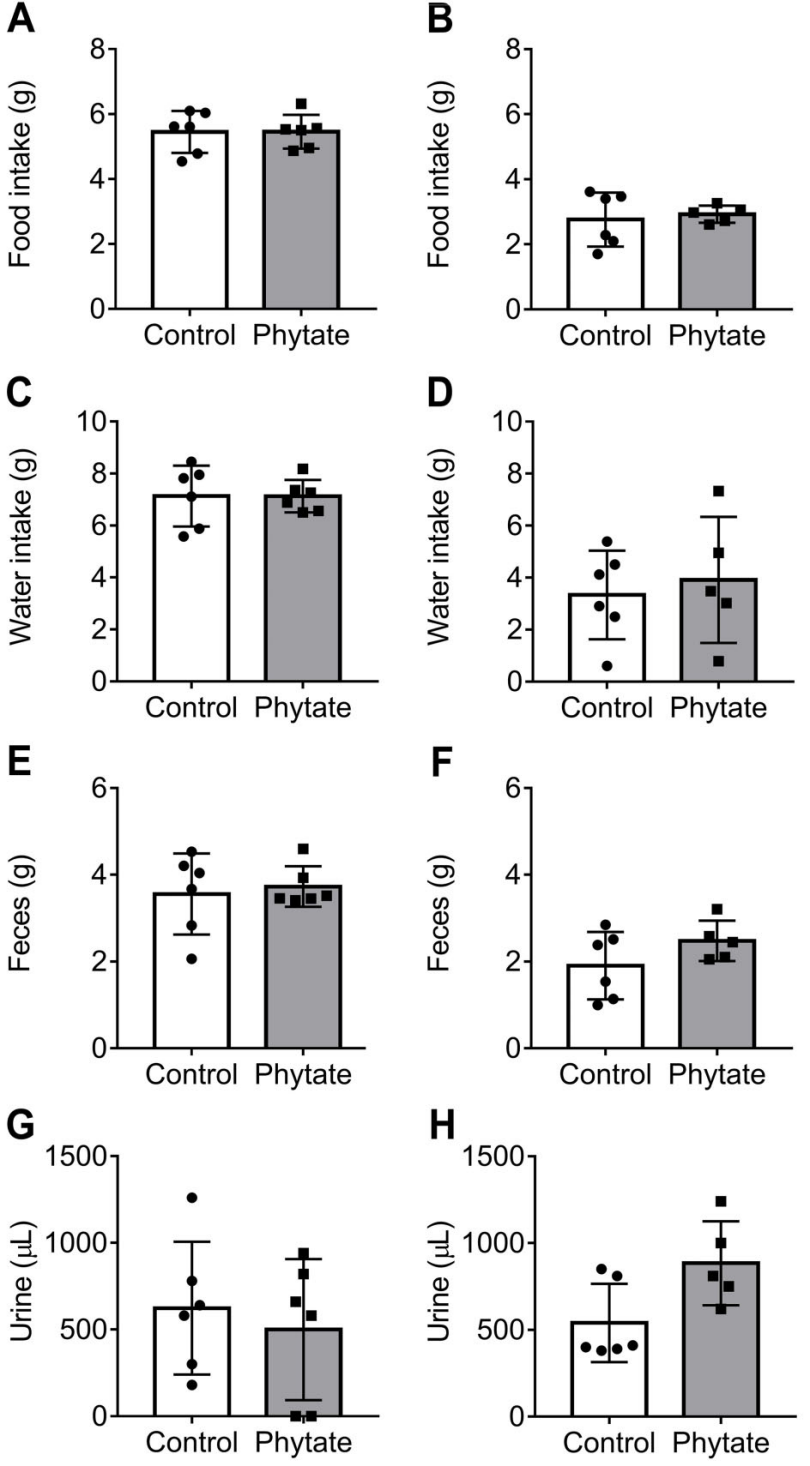
Metabolic assessment. Twelve-hour food intake (**A** and **B**), water intake (**C** and **D**), and fecal (**E** and **F**) and urine (**G** and **H**) of animals consuming control or phytate-supplemented diets were evaluated in the 3rd (**A**, **C**, **E**, **G**) and 6th (**B**, **D**, **F**, **H**) week of intervention. The data are reported as means±SD. P>0.05, *t*-test.

We found a higher content of sodium (21%) and a modest increase in phosphorous (8.3%) in the intervention group. No conclusion, however, can be drawn from these results alone. No difference in food consumption and water ingestion was observed between the control and intervention groups (Figure 2). Feces and urine production was also similar between groups (Figure 2). In conjunction, these data indicated that the addition of phytate to the diet did not alter nutrient ingestion or feces/urine production.

### Metabolic effect of phytate ingestion

**Figure 3:**
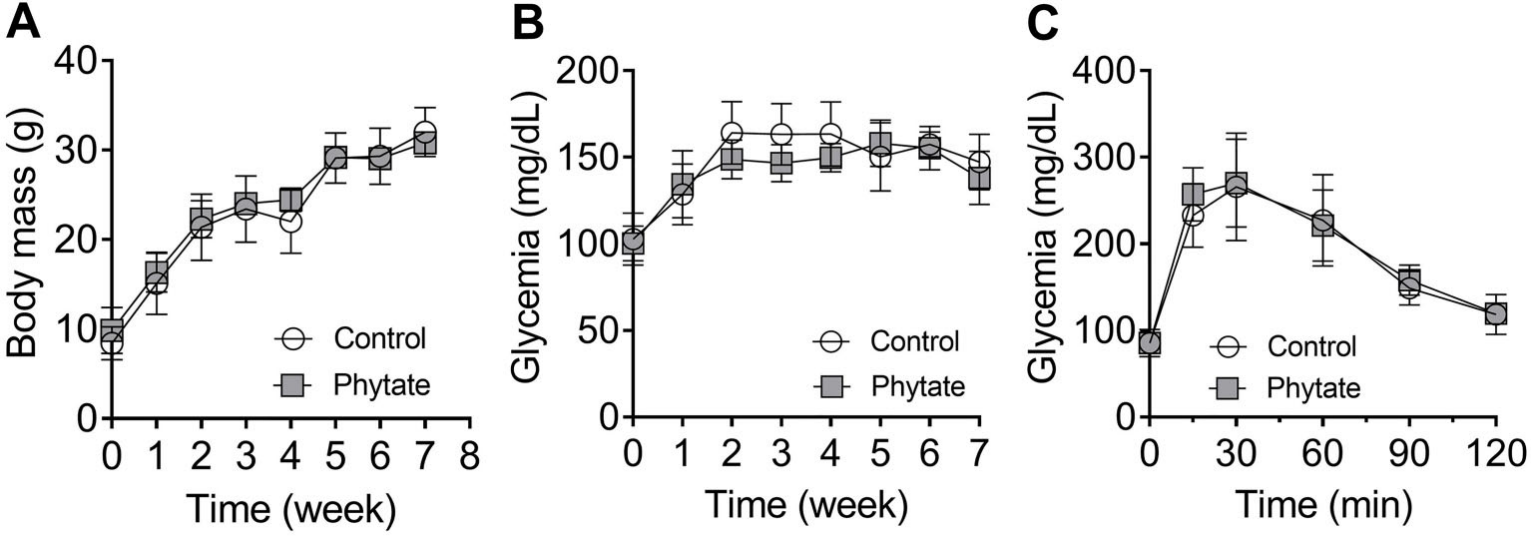
Body weight and glucose metabolism. **A**, body weight curve; **B**, capillary glycemia curve; and **C**, oral glucose tolerance test (OGTT) of control (n=5) and phytate-supplemented (n=6) groups. Data are reported as mean and standard deviation. No significant differences were found between groups by two-way ANOVA.

During the intervention, body weight did not differ significantly (Figure 3A). Glycemic differences were also not found during the intervention, as assessed by random capillary measurements (Figure 3B). Glycemic curves were also similar between control and intervention groups (Figure 3C), suggesting no marked effect of phytate on global glycemic disposal.

**Figure 4:**
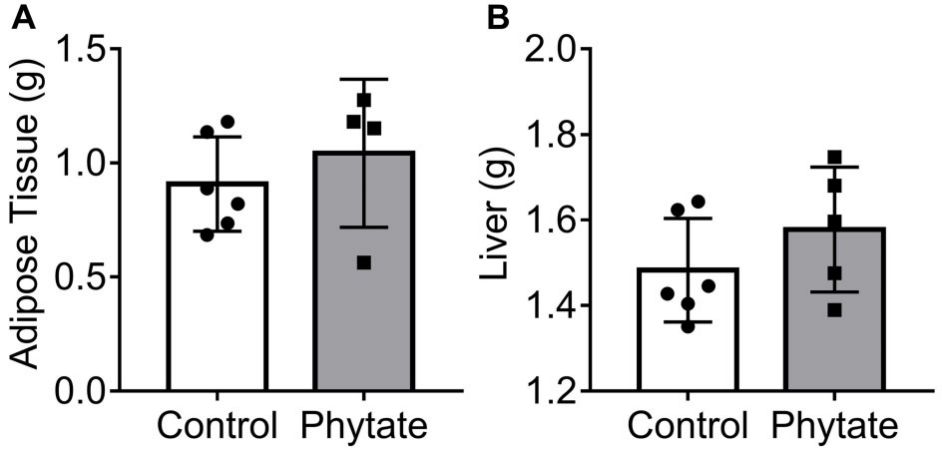
Tissue macroanalysis. **A**, Adipose tissue weight of control (n=6) and phytate-supplemented (n=4) groups. **B**, Liver weight of control (n=6) and phytate-supplemented (n=5) groups. No significant differences were found between groups (Student's *t*-test). Data are reported as as means±SD.

After euthanasia, the abdominal adipose tissue (Figure 4A) and liver (Figure 4B) were collected and measured. No difference in the size of these organs was found between the control and intervention groups.

**Figure 5:**
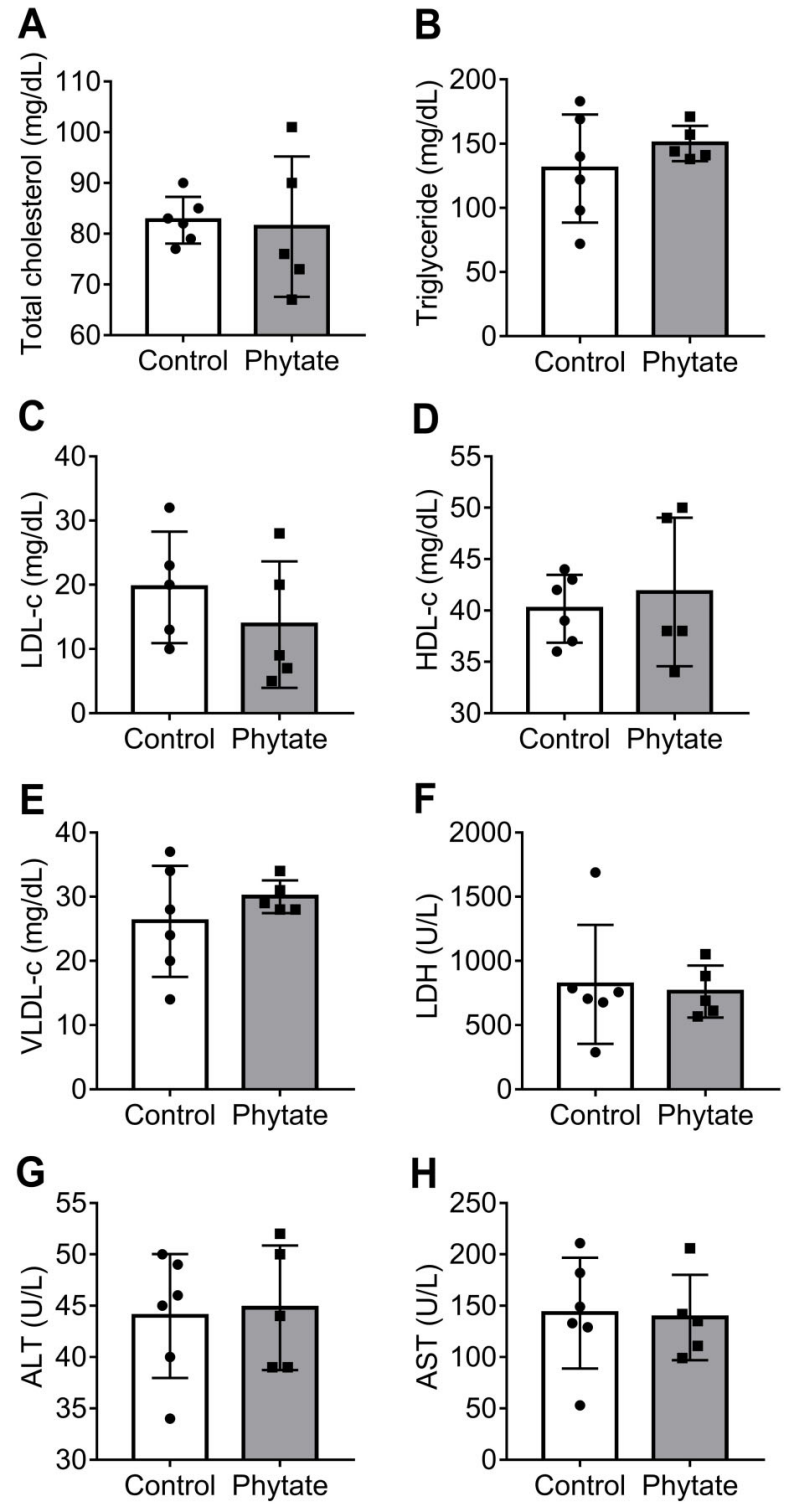
Biochemical analysis. **A**, Total cholesterol; **B**, triglycerides; **C**, low-density lipoprotein cholesterol (LDL-c); **D**, high-density lipoprotein cholesterol (HDL-c); **E**, very-low-density lipoprotein cholesterol (VLDL-c); **F**, lactate dehydrogenase (LDH); **G**, alanine aminotransferase (ALT); and **H**, aspartate aminotransferase (AST) analysis of control (n=5) and phytate-supplemented groups (n=6). No significant differences were found between groups for each analysis (Student's *t*-test). Results are reported as means±SD.

A biochemical analysis of the plasma also showed no major differences between the groups in liver function and damage, as judged by the levels of AST, ALT, and LDH (Figure 5). These data indicated that dietary phytate did not induce major hepatic injuries and function disruption, as judged by the size of liver and abdominal adipose tissue.

### Effect of dietary phytate on nociceptive pain

**Figure 6:**
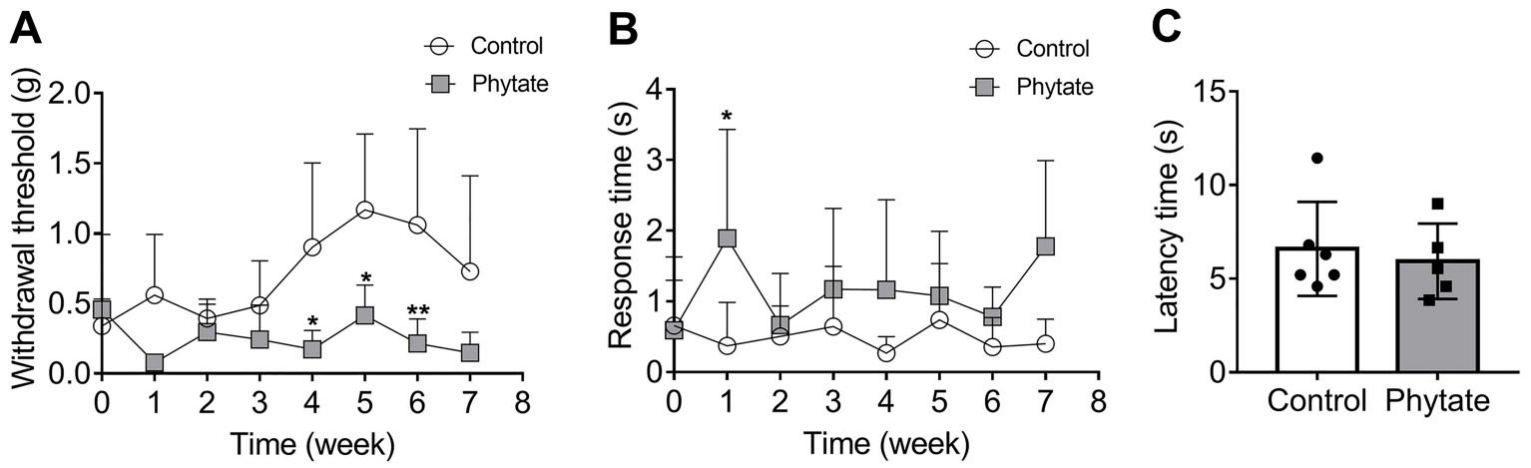
Paw sensitivity. Animals from the control and phytate-supplemented groups were evaluated for their mechanical threshold with von Frey filaments (**A**), cold allodynia by acetone test (**B**), and heat sensitivity (**C**). Data are reported as mean and standard deviation (n=5-6). *P<0.05 and **P<0.01 compared to control by two-way ANOVA followed by Sidak's multiple comparison post-test (**A** and **B**) and Mann Whitney test (**C**).

To evaluate the potential effect of dietary phytate on mechanical sensitivity, animals were subjected to weekly measurement by the von Frey test. The control group showed a progressive increase in the paw withdrawal threshold, as usually expected in the growing phase (Figure 6A). However, animals receiving dietary phytate showed a lower threshold, which was almost constant compared to baseline upon weaning.

The cold test showed a significant difference (P<0.05) only in the first week of the intervention, with no further differences between groups (Figure 6B). The Hargreaves heat test also showed no difference between groups (Figure 6C). Collectively, these data indicated a specific effect of dietary phytate in the induction of nociceptive pain.

### No major effect of dietary phytate on locomotion and cognition

**Figure 7:**
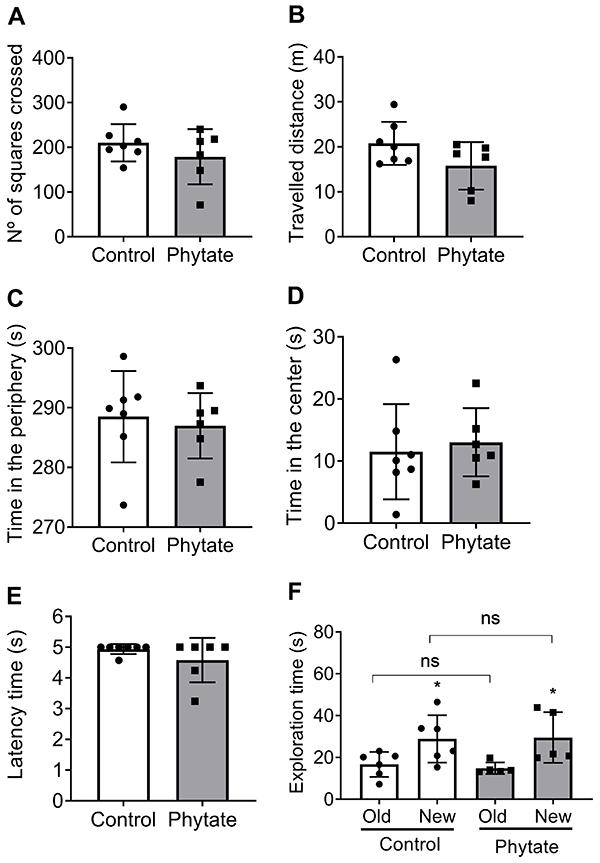
Motor activity and cognition of animals receiving control and phytate-supplemented diets. Motor activity was evaluated by the open field test evaluating number of squares crossed (**A**), total travelled distance (**B**), time in the periphery (**C**), time in the center (**D**), and by the rota-rod test (**E**). The declarative memory was accessed by the object recognition test (**F**). No significant differences were found between groups (P>0.05; Student's *t*-test). ns: not significant.

The open field test was performed in the 5th week of intervention, where metabolic changes were already installed in the animals, as suggested by the von Frey test (Figure 6A). The intervention group receiving supplemented phytate showed no difference from the control group in the number of crossing (Figure 7A) and total distance (Figure 7B). No differences were also observed in the latency time in the periphery (Figure 7C) and in the time in the center of the open field (Figure 7D), suggesting no locomotion effect of dietary phytate. The rota-rod test results for locomotion, cognition, and strength were also not significantly different between the groups (Figure 7E). Since no motor disorder was observed, we subjected both groups to a robust cognition evaluation. The object recognition test conducted in the 5th week of intervention showed no difference in exploratory behavior of the animals receiving dietary phytate compared to the control group (Figure 7F). Collectively, these data supported the lack of generalized motor or cognitive disorder in the intervention group compared to the control.

### TNF level in spinal cord was reduced due to dietary phytate

**Figure 8:**
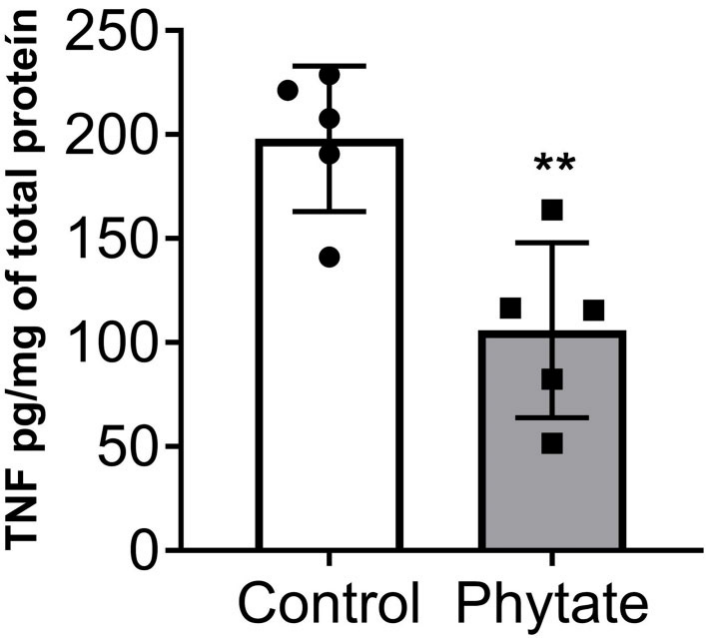
Spinal cord tumor necrosis factor (TNF) level of control and phytate-supplemented groups. Spinal cord was collected eight weeks after dietary intervention. Data are reported as means±SD (n=5). **P<0.01 (Student's *t*-test).

To gain insight into the mechanisms surrounding nociceptive pain, the levels of TNF in the spinal cord were evaluated. Mice receiving a diet supplemented with phytate presented a lower level of TNF compared to control (Figure 8). These data demonstrated that dietary phytate lead to modulation of the inflammatory pathway involved in nociceptive pain, although it did not sufficiently alter overall energy metabolism in mice.

## Discussion

Phytate is known to chelate and consequently reduce the bioavailability of some micronutrients, especially divalent metals such as iron, zinc, calcium, and manganese, whose absorptions are affected by phytate. Removal of phytate enhances the bioavailability of these cations, and several methods are used in the food industry to remove it in order to improve the nutritional value of grains, widely used in the human diet worldwide (23,24).

In the present work, we found that dietary phytate resulted in significant reduction in mechanical allodynia threshold associated with a lower inflammatory marker within only 5 weeks of intervention, without further macronutrient alterations, motor development, memory, and painful processes.

Dietary phytate at 10% has been shown to decrease food intake, affecting rodent growth, and induce dyslipidemia, while lower levels induced non-significant effects on food intake and weight gain (25). Since decreased food intake could result in confounding factors beyond micronutrient bioavailability, we used 1% phytate in the present study.

Regarding total absorption of minerals *in vivo*, the amounts of sodium and phosphorus were higher in the feces of animals in the supplemented diet group (20.8 and 8.5%, respectively), corroborating data found in the nutrient content analysis of the modified diet. The higher sodium level can be explained by the addition of sodium phytate and the higher phosphorus content by the phytic acid molecule itself, which is known to be the main phosphorus storage compound in grains and seeds (24).

Body weight and random blood glucose of the animals were monitored weekly and the glucose tolerance test was performed at the end of the experiment. No differences in these parameters were observed between groups. It should be noted that our study was carried out in healthy and growing animals without any previous disease and limited to the observation of changes caused specifically by the consumption of phytate through the diet. In contrast, studies in rats submitted to the streptozotocin-induced diabetes model show that phytic acid supplementation for 30 days leads to an increase in food intake, a decrease in body weight gain, and a reduction in the percentage of random peak blood glucose in these animals (26). Our results also showed that there was no dietary change caused by phytate supplementation, given the data on water and food intake and urinary and fecal excretion.

Some studies report a lipid-lowering effect of phytate. Onomi et al. (25) observed that the addition of phytate, even at a very low concentration (0.02%), was able to significantly reduce the hepatic levels of cholesterol, triglycerides and lipogenic enzymes (fatty acid synthase, malic enzyme, and glucose-6-phosphate dehydrogenase) in a diabetic rat model. However, the significant differences were observed only in serum lipid levels from higher concentrations (5 and 10%). Corroborating these findings, we also did not observe significant differences in serum lipid levels in the presence of 1% phytate.

Omoruyi et al. (26) evaluated the levels of total cholesterol, HDLc fraction, and triglycerides after 4% phytate supplementation in diabetic rats and found no differences in the serum lipid profile of these animals. The same authors analyzed liver and kidney weight and found no differences, as in the present study, without changes in liver and adipose tissue weight.

However, the transcription of the TNF marker seems was decreased in the phytate group. Phytate modulates the transcription of genes encoding TNF and its receptors (TNFRI and TNFRII) in human colon cancer cells line (Caco-2), where the transcription of TNFRI is increased and of TNF and TNFRII reduced, showing an anti-inflammatory and antitumor role of phytate (27). Data from our group also demonstrated a reduction of this cytokine in the plasma of animals submitted to a zinc-deficient diet (6). Likewise, in our experiments, we observed a decrease in the levels of TNF expressed in the spinal cord of mice that received phytate.

Phytate supplementation has been shown to alter the levels of some pro-inflammatory markers. Diabetic rats that received 4% phytate supplementation for 30 days showed elevated plasma levels of IL-1β (26).

Before evaluating any behavioral parameters, such as pain response and cognition, it is important to determine if the mouse's motor capacity is not compromised, as tasks that require strength and motor coordination may have impaired results. To this end, motor coordination has traditionally been assessed by the rota-rod test and cognition by the object recognition test (10,16,28). In our studies, we did not observe impairments in basic motor functions nor in the short-term memory in the phytate group.

Mechanical allodynia is generally associated with nerve damage, and several central and peripheral mechanisms are implicated in its establishment, including phenotypic changes in peripheral and central neurons and as an important role for immune cells (2,29). In this sense, good neuronal development is essential to prevent the onset of chronic pain. Therefore, tissues require adequate amounts of micronutrients, and dietary intake must be sufficient to meet these needs and maintain health. In addition to optimal bioavailability, it is important to provide adequate conditions for the availability of these nutrients in the diet, especially mineral micronutrients (30-32).

In our study, the presence of phytate in the diet of animals after weaning led to the development of a persistent mechanical allodynia during the growth of these animals. A limitation in the present study is that the mechanism involved in this phenomenon is unknown. Whether this is a direct effect of daily consumption of phytate or whether it results from changes in the bioavailability of divalent metals, essential and important for neurodevelopment, needs further investigation. Divalent metals such as Zn, Mg, Ca, and Cu have already been described and implicated as mitigating agents in other pain models (4,6,33-37). Moreover, the effect of adding sodium ions to the diet should also be considered, which is difficult to consider as a single nutrient. Further studies are needed to elucidate the mechanisms behind these nociceptive and neuronal responses induced by dietary phytate supplementation in healthy animals.

It remains unclear which element(s) may be responsible for the observed effects of phytate supplementation. Since phytate chelates bivalent ions rather indiscriminately, it leaves open multiple possibilities for potential targets and thus acts by multiple mechanisms, as suggested by the increased iron uptake (and lower fecal iron content) and no effect on body weight (in contrast to dietary zinc restriction in the absence of phytate). This suggested that phytate also acts by mechanisms independent of its metal-binding capability.

In conclusion, the consumption of dietary phytate for 8 weeks, included in the chow at 1% w/w, was sufficient to promote subclinical mechanical allodynia along with a reduction in an inflammatory biomarker in the spinal cord. These data indicated that phytate may promote a silent disruption of the neuronal system without significant alteration of major metabolic and biologic biomarkers.
